# G-quadruplex conformation and dynamics are determined by loop length and sequence

**DOI:** 10.1093/nar/gku464

**Published:** 2014-06-11

**Authors:** Ramreddy Tippana, Weikun Xiao, Sua Myong

**Affiliations:** 1 Bioengineering Department, University of Illinois, 1304 W. Springfield Ave., Urbana, IL 61801, USA; 2 Biophysics and Computational Biology, 1110 W. Green St., Urbana, IL 61801, USA; 3 Institute for Genomic Biology, 1206 Gregory Drive, Urbana, IL 61801, USA; 4 Physics Frontier Center (Center of Physics for Living Cells), University of Illinois, 1110 W. Green St., Urbana, IL 61801, USA

## Abstract

The quadruplex forming G-rich sequences are unevenly distributed throughout the human genome. Their enrichment in oncogenic promoters and telomeres has generated interest in targeting G-quadruplex (GQ) for an anticancer therapy. Here, we present a quantitative analysis on the conformations and dynamics of GQ forming sequences measured by single molecule fluorescence. Additionally, we relate these properties to GQ targeting ligands and G4 resolvase 1 (G4R1) protein binding. Our result shows that both the loop (non-G components) length and sequence contribute to the conformation of the GQ. Real time single molecule traces reveal that the folding dynamics also depend on the loop composition. We demonstrate that GQ-stabilizing small molecules, *N*-methyl mesoporphyrin IX (NMM), its analog, NMP and the G4R1 protein bind selectively to the parallel GQ conformation. Our findings point to the complexity of GQ folding governed by the loop length and sequence and how the GQ conformation determines the small molecule and protein binding propensity.

## INTRODUCTION

Although the G-quadruplex (GQ) has long been thought to be an *in vitro* artifact, numerous recent studies point to the existence of GQ *in vivo*. Bioinformatics studies located GQ forming sequences in functional regions of the genome such as the transcription start site and telomeres, suggesting a potential regulatory role (1–4). GQ-binding ligands show that GQs located near promoter regions are directly involved in transcriptional regulation (5–7). Additionally, a recent genome-wide deep sequencing study identified that origins of replication are significantly associated with the GQ motifs (2). Moreover, the failure to resolve these structures induce genomic instability, further supporting the formation of GQ structures *in vivo* (1). GQs have been explicitly implicated in disease onset. The stable GQ structure that arises from the hexanucleotide repeat expansion (HRE), (GGGGCC)*_n_*, was reported to be the most common genetic cause of the neurodegenerative diseases such as amyotrophic lateral sclerosis (ALS) and frontotemporal dementia (FTD) (8,9). In mRNA, GQs have shown a translation repression of a virus (8).

Guanine rich single stranded DNA has a strong propensity to fold into GQ *in vitro*. The basic formula of [G_3_N_1–7_G_3_N_1–7_ G_3_N_1–7_ G_3_] allows four sets of G triplets to form into three layers of G tetrads, mediated by the Hoogsteen base pairing (10). The GQ structures are stabilized by monovalent cations such as potassium or sodium. These ions occupy the central cavity created by the stacks of G tetrads (11–13). GQ DNA can fold into parallel, antiparallel and hybrid conformations depending on its loop length and sequence composition (14). Conventional techniques such as circular dichroism (CD) and thermal melting curves acquired through UV–visible spectroscopy are often used to distinguish GQ folding into parallel and antiparallel conformations (15). CD readings will provide either a characteristic peak at 260 nm for parallel or 295 nm for the antiparallel state. This allows for qualitative comparison among various GQ forming sequences (16). As demonstrated before, the single molecule FRET (smFRET) technique offers several advantages over ensemble methods. First, the fraction of molecules that fold into different conformations (parallel and antiparallel) can be quantified with accuracy. Second, unfolded DNA can be distinguished from folded conformations. Third, the real-time imaging of single molecules allows for the monitoring of molecules undergoing transitions from one state to another, thus enabling kinetic analysis. This approach was applied in studies of telomeric DNA (17–19), modified GQ sequences in various solution conditions (18), GQ binding ligands (20) and protein interactions with the telomere overhang (21–23).

By using the smFRET assay developed previously (17,24), we show that the conformations of GQ are modulated by the loop length and sequence. We confirm that smaller loops promote a more parallel GQ structure, which agrees with previous reports (25,26). Additionally, our results reveal new insights about the GQ folding patterns which is modulated by the length and composition of the loop sequence. We present a systematic analysis of GQ conformation and dynamics governed by loop length and nucleotide composition and how these properties may modulate loading of small molecules and proteins. Our results can provide a useful reference for assessing the GQ folding potential of various important genetic elements including promoter sequences.

## MATERIALS AND METHODS

### DNA sample preparation

All oligonucleotides required to GQ DNA substrates were purchased from IDT with either Cy3 or Cy5 dyes (Table 1). The complimentary DNA was modified with an amino modified C6 dT, eight bases from the 5′ end and reacted with NHS-ester conjugated Cy5 (GE Healthcare). Ten millimolars dye was incubated with 0.1 mM DNA in 100 mM sodium tetraborate pH 8.5 buffer over 4–5 h. The excess dye was removed using Micro Bio-spin 6 column (Bio-Rad) twice. All GQ DNA constructs were annealed by mixing the 3′Cy3 GQ containing DNA and the complementary Cy5 labeled-3′ biotinylated DNA at a molar ratio of 1:1.5 in T50 (10 mM Tris–HCl pH 7.5, 50 mM NaCl). The annealing reaction was performed by incubating at 95°C for 2 min then slowly cooling to room temperature for 2 h.

**Table 1.** DNA oligonucleotide for GQ constructs

Name	Sequence 5′ to 3′
cMyc	TGG CGA CGG CAG CGA GGC GGG T GGG GA GGG T GGG/3′Cy3
133	TGG CGA CGG CAG CGA GGC GGG T GGG TTT GGG TTT GGG/3′Cy3/
144	TGG CGA CGG CAG CGA GGC GGG T GGG TTTT GGG TTTT GGG/3′Cy3/
155	TGG CGA CGG CAG CGA GGC GGG T GGG TTTTT GGG TTTTT GGG/3′Cy3/
177	TGG CGA CGG CAG CGA GGC GGG T GGG TTTTTTT GGG TTTTTTT GGG/3′Cy3/
199	TGG CGA CGG CAG CGA GGC GGG T GGG TTTTTTTTT GGG TTTTTTTTT GGG/3′Cy3/
233	TGG CGA CGG CAG CGA GGC GGG TT GGG TTT GGG TTT GGG/3′Cy3/
333(TTT)	TGG CGA CGG CAG CGA GGC GGG TTT GGG TTT GGG TTT GGG/3′Cy3/
433	TGG CGA CGG CAG CGA GGC GGG TTTT GGG TTT GGG TTT GGG/3′Cy3/
533	TGG CGA CGG CAG CGA GGC GGG TTTTT GGG TTT GGG TTT GGG/3′Cy3/
TTA	TGG CGA CGG CAG CGA GGC GGG TTA GGG TTA GGG TTAGGG/3′Cy3/
TAA	TGG CGA CGG CAG CGA GGC GGG TAA GGG TAA GGG TAA GGG/3′Cy3/
AAA	TGG CGA CGG CAG CGA GGC GGG AAA GGG AAA GGG AAA GGG/3′Cy3/
T25	TGG CGA CGG CAG CGA GGC (T)_25_/3′Cy3/
Amino 18 nt	GCC TCG C/iamino/TG CCG TCG CCA/3′Bio/(annealed to all the 3′ Cy3 sequence listed above)
313	TGG CGA CGG CAG CGA GGC GGG TTT GGG T GGG TTT GGG
331	TGG CGA CGG CAG CGA GGC GGG TTT GGG TTT GGG T GGG
515	TGG CGA CGG CAG CGA GGC GGG TTTTT GGG T GGG TTTTT GGG
551	TGG CGA CGG CAG CGA GGC GGG TTTTT GGG TTTTT GGG T GGG
717	TGG CGA CGG CAG CGA GGC GGG TTTTTTT GGG T GGG TTTTTTT GGG
771	TGG CGA CGG CAG CGA GGC GGG TTTTTTT GGG TTTTTTT GGG T GGG
919	TGG CGA CGG CAG CGA GGC GGG TTTTTTTTT GGG T GGG TTTTTTTTT GGG
991	TGG CGA CGG CAG CGA GGC GGG TTTTTTTTT GGG TTTTTTTTT GGG T GGG

### Single molecule imaging buffers

For single molecule imaging, 0.8 mg/ml glucose oxidase, 0.625% glucose, ∼3 mM 6-hydroxy-2,5,7,8-tetramethylchromane-2-carboxylic (Trolox), and 0.03 mg/ml catalase were added to the buffer (10 mM Tris–HCl pH 7.5 with or without 100 mM KCl). All CD measurements were carried out in the same basic buffer (10 mM Tris–HCl pH 7.5 and 100 mM KCl) at room temperature (23 ± 1°C).

### Single molecule fluorescence data acquisition

Single molecule fluorescence experiments utilized quartz slides (Finkenbeiner) coated with polyethylene glycol (PEG) as described previously (27). Briefly, the slides and coverslips were cleaned with combination of methanol, acetone, potassium hydroxide and flame treatment. These slides were then coated with aminosilane followed by a mixture of 97.5% mPEG (m-PEG-5000, Laysan Bio, Inc.) and 2.5% biotin PEG (biotin-PEG-5000, Laysan Bio, Inc).

The annealed DNA molecules were immobilized on the PEG-passivated surface via biotin–neutravidin interaction. All experiments and measurements were carried out at room temperature (23 ± 1°C). Prism type total internal reflection microscopy was used to acquire single molecule FRET. A 532-nm Nd:YAG laser was guided through a prism to generate an evanescent field of illumination (27). Data was recorded with a time resolution of 100–200 ms and analyzed with custom scripts written in interactive data language (IDL) to give fluorescence intensity time trajectories of individual molecules.

### smFRET data analysis

Basic data analysis was carried out by scripts written in Matlab, with FRET efficiency, *E*, calculated as the intensity of the acceptor channel divided by the sum of the donor and acceptor intensities. FRET histograms were generated using over 6000 individual molecules and were fitted to Gaussian distributions using Origin 8.0 (peak position left unrestrained). Dwell times were collected by measuring the time that each molecule spends in a particular FRET state. The means and the standard errors were plotted. Software for analyzing single-molecule FRET data is available for download from https://physics.illinois.edu/cplc/software/.

### Circular dichroism

The CD spectra were recorded at room temperature (23 ± 1°C) on a JASCO J-715 spectropolarimeter over the range of 200–320 nm using a 1-mm path length quartz cuvette with a reaction volume of 200 μl. The GQ oligonucleotides concentration was 15 μM.

### Steady state quenching measurements

Bulk fluorescence quenching measurements were performed at room temperature in a standard buffer condition (10 mM Tris–HCl pH7.5, 100 mM KCl) with 50 nM of the previously mentioned GQ DNA minus the 3′ biotin. Cy3 Fluorescence excitation was set at 532 nm and emission was monitored at 572 nm. Bandwidths of both excitation and emission filter set at 10 nm. Fluorescence quenching was initiated with the small amount of GQ ligand and monitored with a fluorescence spectrophotometer (Cary Eclipse, Varian). Fluorescence quenching curves were fitted with a double exponential fit to establish a saturation point. Additionally, the hill coefficient was calculated for the quenching curves to determine the *K*_d_ of each drug binding (shown below). The ligands utilized (NMM, NMP and NMMDE) were purchased from Frontier Scientific, Inc., UT, USA:

\begin{equation*} y = V_{\max } \frac{{x^n }}{{x^n + k^n }}, \end{equation*}

where *y* is the percentage of fluorescence quenching and *x* is the small molecule drug concentration.

### G4 resolvase 1 purification

Codon optimized cDNA of G4 resolvase 1 was purchased from GeneScript, Inc., NJ, USA. The cDNA was transformed into the BL21(DE3) *Escherichia coli* strain. Cells were grown at 37°C until OD (optical density) reached 0.6. Then 0.6 mM IPTG (isopropyl-beta-D-thiogalactopyranoside) was added to the *E. coli* culture for induction and it was kept 14°C for overnight to reach OD of 1.2. The rest of protein purification followed previously published protocol with minimal changes (28). C-MYC G4-DNA bound streptavidin paramagnetic beads (CGSPB) were prepared by adding to 3 OD of biotin c-MYC 51mer (Supplementary Table S1) DNA to 2 ml of MagnaBind magnetic beads from Thermo Scientific, USA. Recombinant G4R1 protein was initially purified by means of a His_6_ tag by utilizing the TALON cobalt beads and xTractor kit according to manufacturer's (Clontech) instructions with 2× Sigma protease inhibitor mixture, 0.01 mM PMSF(phenylmethylsulfonyl fluoride) and 15 μg/ml leupeptin added. BL21cell lysates were isolated and bound to TALON cobalt (0.5 ml bead volume per 500 ml of *E. coli* culture) resin as recommended by the manufacturer. Cobalt resin was washed three times with ice-cold SSC (4×) with β-mercaptoethanol (0.5 μl/ml). Recombinant protein was eluted from resin with three washes of 0.5 ml of histidine elution buffer (0.7 M histidine, pH 6.0, 8.6 mM β-mercaptoethanol, 1× Sigma protease inhibitor mixture), followed by one 0.5-ml wash of 200 mM EDTA(Ethylenediaminetetraacetic acid) pH 6.0. For the second phase of purification, the four elutes were combined with 1 ml (3 ml total) of 3× Res buffer (1×, 50 mM Tris acetate, pH 7.8, 50 mM NaCl, 70 mM glycine, 0.5 mM MgCl_2_, 0.012% bovine α-lactalbumin, 1× Sigma protease inhibitor mixture, 10% glycerol) and bound to CGSPB at 37°C for 15 min. Bound CGSPB (C-MYC G4-DNA bound streptavidin paramagnetic beads) were washed two times in ice-cold SSC (4×) with 0.1% α-lactalbumin and 0.5 μl/ml β-mercaptoethanol. High purity recombinant His-tagged G4 resolvase 1 was obtained by ATP-dependent elution of CGSPB as described previously (29) except bovine α-lactalbumin and Sigma protease inhibitor mixture were added to the elution buffer. Purified enzyme stock was stored at –80°C.

### Electrophoretic mobility shift assay (EMSA)

Ten nanomolars partial duplex GQ containing the Cy5 dye at junction (Supplementary Table S1) were mixed with 10 nM of the G4R1 and incubated for a short time (3 min) in buffer containing 10 mM Tris–acetate pH 7.8, 50 mM KCl, 50 mM NaCl, 0.5 mM MgCl_2_ and 10% glycerol. The reaction mixture was loaded and run on a 6% acrylamide gel at 65 V for 2 h with 0.5× TBE (Tris Borate EDTA) running buffer. Gel images were taken with ImageQuant LAS4010 imager from GE (General Electric). Analysis in ImageJ was used to quantify the percentage binding by taking the area of shifted band corresponding to G4R1 bound DNA and dividing it by the total area sum of DNA with G4R1 and DNA.

## RESULTS

### GQ folding conformation analyzed by FRET

**Figure 1.**
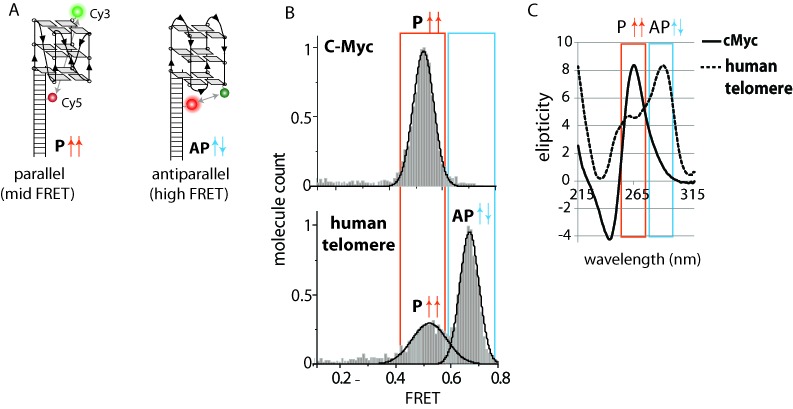
G-quadruplex conformation is distinguished by FRET. (**A**) The GQ containing overhang DNA with Cy3 (green) dye at 3′ end and Cy5 (red) dye. (**B**) FRET histograms for c-Myc and human telomere DNA. (**C**) CD spectrum of c-Myc and human telomere DNA.

A 18-bp partially duplexed DNA with Cy3 (green) dye at the 3′ end of the ssDNA overhang and Cy5 (red) dye at the eighth position (from the junction) of the complementary strand was utilized to monitor GQ folding (Figure 1A). The specific position of the two dyes was chosen to be sensitive to the differences in GQ folding attributed to parallel (F1), antiparallel (F2) and unfolded states (UF) (17,21,24). When in parallel, all four G-triplets are expected to point in the same direction (upward as drawn), resulting in a mid-FRET value (∼0.55) due to the separation between the two dyes. Since the hybrid structure can also give rise to the mid-FRET value, CD measurements were utilized to further distinguish the different folding configurations. In the antiparallel case, the G-triplets will alternate in directionality, which will yield high FRET (∼0.7) due to the resulting proximity between the two dyes (Figure 1A).

c-Myc and the human telomeric sequence were utilized to test if our assay allows for distinguishing parallel from antiparallel GQ folding (Table 1). The c-Myc sequence is known to only fold into a parallel configuration in 100 mM KCl (30,31). We applied the c-Myc DNA labeled with Cy3 and Cy5 (Figure 1A) to single molecule imaging surface for FRET detection using total internal reflection microscope. (19,32). One field of view yields ∼300–400 single molecules that gives FRET value. The FRET histogram was built from the FRET values collected from over 1000 DNA molecules derived from three to four areas of the imaging surface. As expected, due to the parallel folding structure of c-Myc, a single peak was observed, centered at 0.55 FRET (Figure 1B, top). In addition, the CD spectrum of c-Myc shows a clear positive peak at 260 nm and a negative peak at 240 nm, which is a signature of parallel GQ folding (33,34) (Figure 1C, orange). The 0.55 FRET value coupled with the CD data suggest that c-Myc is folded in parallel configuration. The CD spectrum of the hybrid GQ is expected to show two peaks at 270 and 290 nm (35). This is significantly different from the 260 nm peak and 240 nm valley that we observe for c-Myc. This verifies that the midFRET peak represents parallel, not a hybrid conformation. This criteria is applied for the analysis of other GQ conformations below.

The human telomere overhang GGG(TTAGGG)_3_ folds into mixed parallel–antiparallel conformations (35). The FRET histogram shows a major peak at 0.7 with a minor peak at 0.55 likely corresponding to the antiparallel and parallel folding, respectively (Figure 1B). The CD data displays a peak at 295 nm with a small shoulder peak at 260 nm, thus suggesting the antiparallel fold as the major conformation (Figure 1D, blue). The complex conformations that can result from the human telomere GQ folding has been reported in numerous structural studies (35–38). Although our results do not pinpoint the exact GQ folding motif, based on the FRET value, CD data and reports from previous studies (17,21), our data is consistent with the parallel (F1, mid FRET) and antiparallel (F2, high FRET) populations. Our assignment of FRET value to the GQ conformation is the same as Ying *et al.* (24) and Lee *et al.* (17), but different from Ray *et al.* (21). This most likely arises from the presence of a flanking DNA sequence utilized in the latter study. The smFRET approach enables us to quantify the individual conformations exhibited by varying GQ forming sequences.

### Loop length-dependent GQ folding

**Figure 2.**
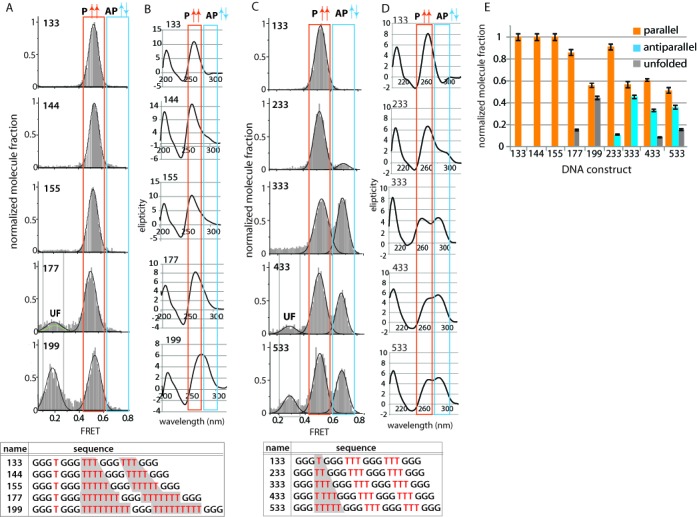
GQ conformation modulated by loop length variation. (**A**) FRET histograms of 133, 144, 155, 177 and 199 DNA. (**B**) CD spectrum of 133–199 DNAs. (**C**) FRET histograms for 133, 233, 333, 433 and 533 DNA. (**D**) CD spectrum of 133–533 DNAs. (**E**) Fraction of parallel, antiparallel and unfolded GQ conformations for all DNAs tested.

Loop length is one of the major determinants of the GQ folding pattern (14). Recently, it has been shown that several G-rich sequences in oncogenic promoters form stable GQ structures (5), often consisting of various lengths of loops. Previous studies have reported the effects of loop length in GQ folding, through ensemble CD, UV melting, fluorescence measurements (12,19,39) and molecular dynamics simulations (40). Here, we systematically varied the loop lengths to quantitatively measure the effects in GQ conformations. Initially, we fixed the first loop at one base and varied the second and third loop from three to nine, notated as 133, 144, 155, 177 and 199, respectively (Figure 2A). The resulting FRET histograms from these constructs show a single peak at 0.55 FRET, indicating a parallel folding conformation. 177 and 199 exhibit an additional lower FRET peak, likely representing an unfolded (UF) population of molecules, consistent with the previous findings (21). CD measurements also agree with these findings, a peak pattern reflecting a parallel folding for all the GQ sequences (sharp peak at 260 nm coupled with a negative peak at 240 nm) is also seen. The peak width is broader for 199, likely affected by the unfolded molecules (Figure 2B). This data set strongly suggests that a single nucleotide in one loop has a dominant effect of inducing a parallel GQ folding. It is surprising that the 199, which exceeds the requirement of [G_3_N_1–7_G_3_N_1–7_ G_3_N_1–7_ G_3_], still folds into parallel, likely governed by the presence of a single base loop (10). Further, the position of the single nucleotide and its propensity to induce parallel folding was explored by moving the single nucleotide to the middle and third loop. In both the middle positions (313, 515, 717 and 919) and third positions (331, 551, 771 and 991), we observe that all DNAs exhibit a clear peak at 260 nm in CD measurement, signifying formation of parallel folding in all DNAs regardless of the single nucleotide position (Supplementary Figure S1).

Next, the number of bases in the first loop was varied from one to five, while keeping three bases in the other two loops. These constructs are named, 133, 233, 333, 433 and 533 (Figure 2C). As we lengthen the first loop from one (133) to two (233) and three (333), a high FRET peak at 0.75 emerged. This indicates the transition from completely parallel to a mixed parallel and antiparallel conformation. To quantify this effect, we calculated the area under the Gaussian fitted curve corresponding to parallel (P), antiparallel (AP) and unfolded (UF) conformations (Figure 2E). Despite one nucleotide difference between 233 and 333, the parallel conformation is significantly higher for 233 (91%) than 333 (56%), suggesting a sharp transition in folding energetics that partitions 233 from 333. Beyond, the 433 and 533 exhibit a mixture of parallel and antiparallel combined with an increasing fraction of unfolded conformation, likely due to the longer loop lengths. We note that the FRET values for the UF population are different due to the total length of single stranded DNA in different DNA constructs. For example, 433 and 533 have total length of 22 and 23 nucleotides (nt) which will yield higher FRET (∼0.3 FRET) values than the 177 and 199 which has 27 and 31 nt (∼0.2 FRET), respectively. CD measurements corroborate with the FRET results. From 133 to 533, a positive peak at 260 nm diminishes while 295 nm peak increases, confirming the decrease in parallel (mid FRET) and increase in antiparallel (high FRET) GQ (Figure 2D). However, the unfolded conformation cannot be deduced from the CD, since it only reports on the existence of parallel and antiparallel state. Taken together, this shows quantitatively how the loop length influences the GQ folding. The longer loop lengths promote antiparallel and unfolded states while the presence of one-base loop dominates the folding into parallel conformation even when neighboring loops are as long as nine bases.

### Loop sequence dependent GQ folding

**Figure 3.**
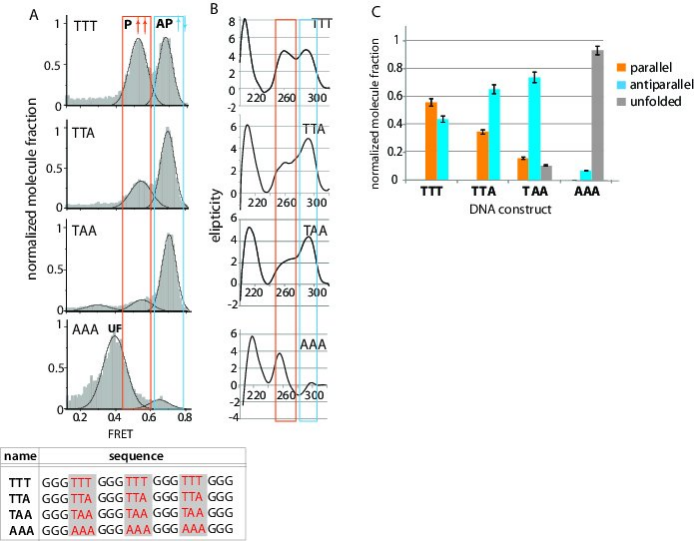
GQ conformation controlled by loop sequence. (**A**) FRET histograms for TTT (333), TTA, TAA and AAA. (**B**) CD spectrum of TTT, TTA, TAA and AAA. (**C**) Fraction of parallel, antiparallel and unfolded GQ conformations for all DNAs tested in (a)

In order to study the role of loop composition in GQ folding, several derivatives of the 333 construct were prepared. Four different sequence compositions (TTT, TTA, TAA and AAA) were tested (Figure 3A). As shown, TTT (333) contains mixture of parallel (56%) and antiparallel (44%) conformations (Figures 2C and 3A). A diminishing population of parallel folding was observed upon adenine (A) replacing thymine (T) in the 333 length constructs. The parallel conformation for TTT, TTA, TAA changed from 56 to 35% and 16%. When all thymines are replaced with adenine as in the AAA construct, a majority of the molecules exhibited a low FRET value corresponding to the unfolded state (Figure 3B). The FRET value for unfolded AAA (0.4 FRET) is higher than that of 433–533 (0.3 FRET) and 177–199 (0.2 FRET). This can be attributed to the shorter ssDNA length of 21 nucleotide as well as a possible helical secondary structure stabilized by ApG or GpA (41). These decreasing parallel population is supported through CD experiments, which show a decrease in the 260 nm peak with increasing adenine content (Figure 3C). The 255 nm peak observed for AAA is likely due to the unfolded GGGAAA repeats (42). This unexpected sequence-dependent effect may be explained by difference in steric hindrance imposed by adenine and thymine in these sequences. Adenine, as a purine, bears two carbon–nitrogen ring unit which is substantially larger than the thymidine with one ring. When in a loop confinement, the adenine bases may experience a greater degree of steric hindrance than the thymines, disfavoring the tight packing of G-triplets in parallel conformation.

### Initial folding conformation and kinetics

**Figure 4.**
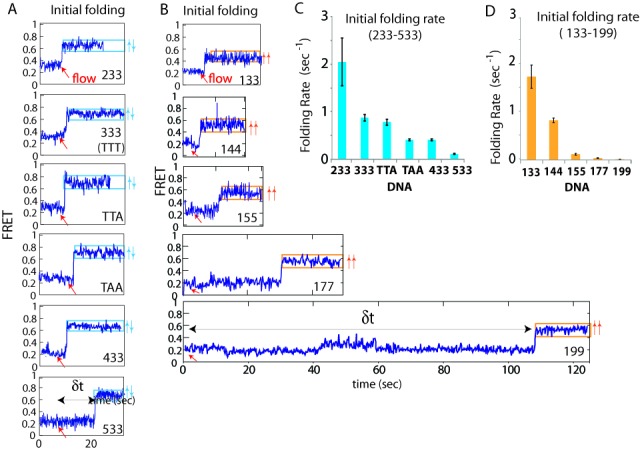
Kinetic analysis of initial GQ folding. (**A**) Representative smFRET traces of initial folding upon KCl addition for 233–533 DNAs. (**B**) smFRET trace of initial folding for 133–199 DNAs. (**C**) Initial folding rate of 233–533 DNAs. (**D**) Initial folding rate of 133–199 DNAs.

Single molecule FRET traces were analyzed for all the constructs tested above in order decipher differences in initial folding and subsequent kinetic behavior. To capture the moment of GQ folding, DNA substrates were incubated in buffer devoid of any cations (10 mM Tris–HCl pH 7.5). This results in no GQ formation. Potassium-containing buffer (100 mM KCl, 10 mM Tris–HCl pH 7.5) was introduced while monitoring the smFRET signal change. Real-time smFRET traces analysis indicated that all sequences initially exhibit low FRET (0.25–0.35), which is expected due to the lack of folding in cation-free buffer conditions. When the potassium buffer is introduced (demarcated by a red arrow), 233–533 constructs initially fold into an antiparallel (0.75 FRET) conformation, while all the one loop length sequences (133–199) fold directly into the parallel state (0.55 FRET) within a time resolution of 100 ms (Figure 4A and B). Initial flow single molecule traces allowed for the calculation of the rate of initial folding by taking the dwell time between the moment of KCl buffer flow and the moment of FRET increase (Figure 4A and B). The results represent a sampling of over 200 molecules for each condition. For 233–533 DNA, the shortest looped constructs, 233 displayed the fastest folding to antiparallel, followed by 333, TTA, TAA, 433 and 533 (Figure 4C). Likewise, 133 exhibited the highest folding rate to parallel conformation followed by 144, 155, 177 and 199. We note that of the rates of short constructs, 133 and 233 are likely underestimated due to the time delay expected from equilibration after the manual buffer flow in our system. This may contribute to increased heterogeneity seen in the folding rates of 133 and 233 (Figure 4C and D). The folding rate between the fastest (133) and slowest (199) differs by more than one order of magnitude according to these measurements. This result signifies that the shorter loop length induces faster folding kinetics.

### Kinetics of conformational exchange

**Figure 5.**
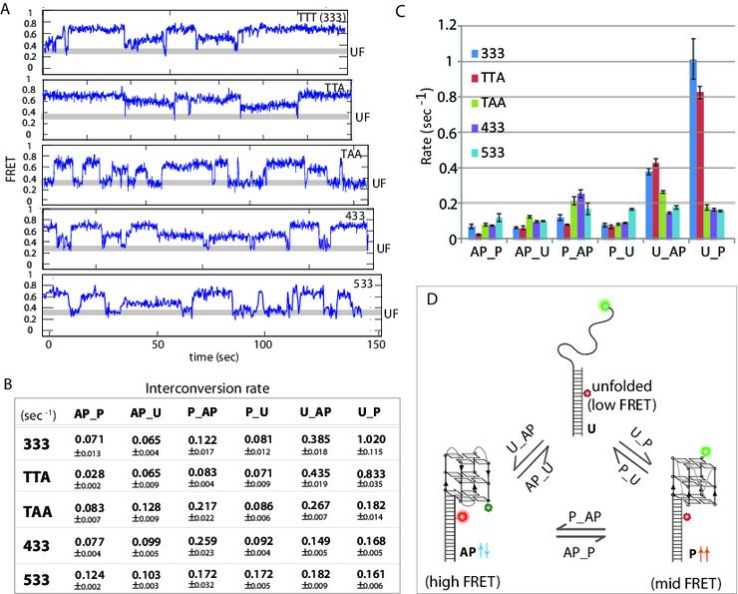
Kinetic analysis of folding-unfolding dynamics of GQ. (**A**) Representative smFRET traces that display folding-unfolding kinetic. (**B**) Kinetic rates of folding and unfolding for all DNAs tested. (**C**) Bar graph of all kinetic rates. (**D**) Diagram showing three state transitions in GQ folding pathway.

After the initial folding, all short looped constructs (133, 233) as well as 144–199 displayed a constant 0.55 FRET, thus, reflecting a stable nature of the parallel folding (Supplementary Figure S2A). Despite the minimal high FRET peak observed for 233 (Figure 2B), the inter-conversion from mid FRET (0.55) to high FRET (0.75) occurs very infrequently, rendering the dwell time collection difficult for these DNAs. In contrast, dynamic folding behavior is observed for TTT (333), TTA, TAA, 433 and 533 constructs (Figure 5A). There are three FRET states in dynamic exchange; antiparallel (0.75), parallel (0.55) and unfolded (0.2–0.3) for all five DNA constructs. The majority of the FRET traces exhibited highly dynamic transitions between all three states. Although, a small fraction of molecules exhibit a long-lived folded states in one specific conformational state (Supplementary Figure S2B). This observation is consistent with the previously reported findings on the human telomere overhang (17) and Tel23 sequence (43). Dwell times of individual FRET transitions representative of the three states and the corresponding six set of kinetic rates were calculated (Figure 5B). For example, the rate at which a parallel state interconverts to an antiparallel state is notated as ‘P_AP’. Similarly, ‘P_U’ refers to the rate at which parallel (mid FRET) folding transitions to an unfolded state (low FRET). Most rates lie within the 0.1–0.2 s^−1^ range (Figure 5C), while substantially faster rates are observed for U_AP and U_P of two constructs, TTA and TTT (333). These rates suggest that TTA and TTT fold into parallel conformation five to six times faster than TAA, 433 and 533. This unusually high folding rate obtained for TTA points to an inherent property built into the human telomeric DNA. This property may have further biological implications.

### Conformation specific binding of NMM, NMP and G4 resolvase 1

NMM (*N*-methyl mesoporphyrin IX) is one of the first small molecules reported to bind GQ DNA (44). Subsequent work has further demonstrated its high specificity for binding GQ DNA (45–47). Such selective binding of NMM to GQ was employed in diverse chemical and biological screening efforts (46,48). NMM binding was also shown to inhibit GQ unwinding by RecQ and BLM helicase (47,49). More recently, a study by Nicoludis *et al.* showed that NMM selectively binds parallel conformation of human telomere sequence (30). We applied NMM and its homolog, NMP to all the GQ DNAs previously investigated to test if their binding propensity displays specificity toward the parallel conformation.

**Figure 6.**
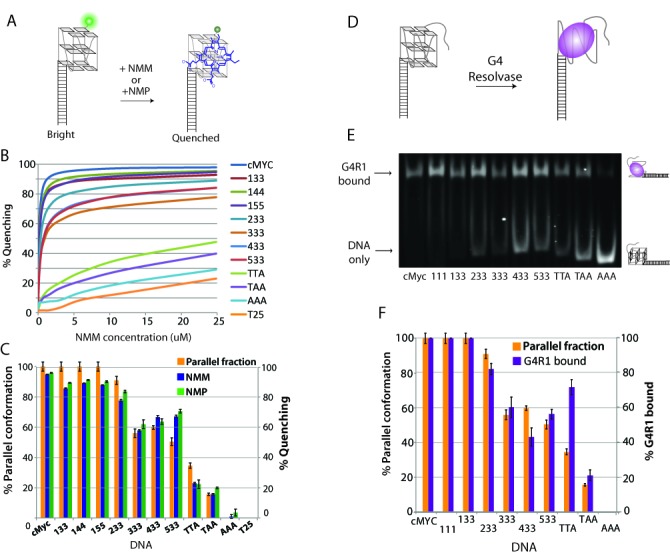
GQ ligand and G4 resolvase binding. (**A**) Fluorescence quenching assay. (**B**) The percentage quenching observed for all GQ DNAs. (**C**) Bar graph plot of percentage parallel conformation (orange) and percentage quenching obtained for NMM (blue) and NMP (green) induced quenching. (**D**) Schematic of G4R1 binding assay. (**E**) EMSA result of G4R1 binding to GQ DNAs. (**F**) Bar graph plot of percentage parallel conformation (orange) and percentage G4R1 binding (purple).

When we applied NMM to GQ DNAs labeled with Cy3 labeled at the 3′ end, we observed immediate quenching of the fluorescence in ensemble measurements (Figure 6A). The quenching level corresponds to the previously observed patterns of parallel GQ conformation. Thus highly parallel sequences exhibited a large degree of quenching, while the highly antiparallel sequences displayed low quenching. We obtained the percentage quenching by taking an inverse of the fluorescence reading as a function of NMM concentration (Figure 6B). As stated previously, most parallel DNAs including c-Myc, 133 and 233 displayed highest degree of quenching whereas the least parallel DNAs such as TTA and TAA exhibited substantially lower quenching. This suggests a selective binding of NMM to parallel GQ. As a negative control, T25 (25 nt deoxy-thymidine) was tested, and this construct resulted in the lowest quenching value. This minimal quenching represents a nonspecific interaction of NMM with the DNA. We performed the same experiment with NMP and obtained quenching signal similar to the NMM (Supplementary Figure S3A). To quantify the parallel specificity of NMM and NMP, we plotted the percentage parallel GQ obtained by FRET (orange) with the maximal percentage quenching by NMM (blue) and NMP (green) after subtracting the T25 signal as a background (Figure 6C). The highly correlated values between the percentage parallel conformation and percentage quenching strongly suggest that NMM and NMP both bind specifically to parallel GQ. From the quenching curve, we obtained the binding dissociation constant, *K*_d_ for NMM, NMP and other GQ ligands including NMMDE and BRACO19 (Supplementary Figure S3B). In agreement with the concentration dependent quenching, NMM and NMP displayed a low *K*_d_ (∼0.1 μM) for highly parallel GQ and high *K*_d_ (10–500 μM) for less parallel GQ constructs. NMMDE and BRACO19 showed less specificity. A macrocyclic drug was also tested which showed substantially less specificity of binding to parallel GQs (Supplementary Figure S3C and D).

The GQ resolving protein, G4 resolvase 1 (G4R1) was tested to establish if any conformation bias exists with respect to which GQ conformation it will bind. The resolvase activity of G4R1 was first identified and characterized by Harrington *et al.* (50) and was later shown to require 3′ ssDNA overhang for loading and unwinding of unimolecular GQs (28). Based on these substrate requirement, we prepared GQ DNA with 15 nt ssDNA overhang to allow G4R1 binding (Figure 6D). We applied purified G4R1 (10 nM) to the GQ DNA constructs and performed EMSA (electrophoretic mobility shift assay). This allowed for the visualization and quantification of protein binding to each GQ sequence (Figure 6E). The band intensity of the G4R1 bound and unbound DNA was quantified allowing for the calculation of bound fraction. The percentage G4R1 binding (purple) was plotted against the percentage parallel GQ (orange) (Figure 6F). This result suggests that G4R1 binding is highly correlated with the parallel percentage, indicating that G4R1 selectively binds parallel GQs.

## DISCUSSION

In this work, we employed the smFRET method not only to distinguish, but to quantify the distinct conformational state that arises from varying loop size and sequence of GQ forming DNA. FRET value alone is not sufficient to report on the exact conformation of GQ folding, thus CD measurement and NMM ligand binding were utilized as complementary results. The added advantage of CD measurement to this method is the distinct signature produced by the parallel GQ conformation that encompasses a sharp peak at 260 nm and a valley at 240 nm. We observed these features for all the GQs that possess single nucleotide loops including c-Myc, 133, 144, 155, 177 and 199. Each of these sequences also displayed a FRET peak at 0.55. The CD spectrum for other GQ folding sequences does not provide a clear observation, as it is difficult to distinguish population distributions when longer loop lengths induce unfolded or less structured GQ molecules.

Real time single molecule FRET traces enabled kinetic analysis of the GQ as they undergo initial folding and the subsequent dynamic behavior. The results suggest that the initial folding rate depends heavily on the overall loop length of the GQ DNA. 133 and 233 which possess seven and eight nucleotides in total loop lengths exhibit ∼2 s^−1^. The total loop size of 9–10 nt shows a substantially reduced rate of 0.5–0.8 s^−1^. When the total loop exceeds 11 nt, the folding rate is diminished to below 0.1 s^−1^. This dramatic decrease in folding rate as a function of total loop length may have implications in the likelihood of folding and unfolding of potential GQ forming sequences in genomic DNA. Short looped GQ may form more readily and persist longer compared to long loop GQs.

The kinetic folding transitions amongst all three conformations (parallel, antiparallel and unfolded) showed interesting relationships. It was observed that the GQ sequences TTT and TTA displayed faster rates of folding when compared to the other GQ sequences tested. In the case of the latter sequence, which is found in human telomeric overhang, this may provide an interesting biological role. The minimization of the time spent in the unfolded state agrees with previous single molecule studies suggesting fast folding kinetics in the context of long telomeric repeats (51). Such a mechanism may help prevent end to end fusion that disrupts the genomic integrity.

Here, we studied human telomere sequence in the context of loop sequence variance. However, human telomere conformation is complex as the structural dynamics are extremely sensitive to its neighboring sequence at either the 3′ or 5′ side (18,36,38). It was also shown to exhibit diverse structures such as hybrid, parallel1, parallel2, basket type structures in the presence of the molecular crowding reagent, PEG (52,53). Here, we focused on the loop size and sequence dependence rather than the complexity arising from the variable sequence arrangement of human telomere.

NMM was shown to bind specifically to parallel GQ formed in human telomeric DNA (31). Upon binding, both the NMM and NMP quenched the fluorescent dye attached to the 3′ end of GQ DNA. This photochemical effect enabled us to quantify the binding propensity and affinity of the ligands. The degree of quenching exhibited for each GQ DNA showed a high correlation to the parallel GQ formation estimated from the FRET histogram analysis. This further demonstrates that the mid FRET level (0.55) that is observed likely represents the parallel fraction of GQ conformation. G4R1 loading preference toward parallel DNA points to the possibility that the parallel GQ may be selectively resolved by an enzyme such as G4R1. Additionally, this suggests that parallel GQs are important structures that require enzymatic resolving in the context of the genome. Additional studies are needed to determine if a helicase is needed to resolve antiparallel GQ conformations. Further, an interesting area has developed in identifying the binding location of G4R1 in genomic DNA in light of the recent finding that human helicases, XPB and XPD, are highly enriched at GQ forming regions near the transcription start site (54).

Our results may be helpful in predicting the possible GQ folding landscape of the promoter. The potential GQ folding sequences in these areas show a large variation in both composition and loop length. Thus, our systematic analysis of composition and loop length dependent properties (folding conformation, initial folding rates and transition kinetics) will prove beneficial in identifying potentially stable folded GQs in this diverse landscape (31,55). The smFRET platform utilized throughout this study can be extended to examine specific promoter sequences and to screen for GQ targeting ligands.

## SUPPLEMENTARY DATA

Supplementary Data are available at NAR Online.

SUPPLEMENTARY DATA
