# Supplementary material for: G-quadruplex conformation and dynamics are determined by loop length and sequence
Source: Nucleic Acids Res. 2014 Jun 11;42(12):8106–14. doi: 10.1093/nar/gku464 (PMC4081081; doi:10.1093/nar/gku464)
Supplement: SUPPLEMENTARY DATA [file supp_42_12_8106__index.html]

G-quadruplex conformation and dynamics are determined by loop length and sequence — SUPPLEMENTARY DATA 

# G-quadruplex conformation and dynamics are determined by loop length and sequence

## SUPPLEMENTARY DATA

**Files in this Data Supplement:**

- Supplementary Figures and Table
