# Supplementary material for: G-quadruplex conformation and dynamics are determined by loop length and sequence
Source: Nucleic Acids Res. 2014 Jun 11;42(12):8106–14. doi: 10.1093/nar/gku464 (PMC4081081; doi:10.1093/nar/gku464)
Supplement: SUPPLEMENTARY DATA [file supp_gku464_nar-01004-f-2014-File008.docx]

**Supplementary Information**

**G-quadruplex conformation and dynamics modulate small molecule and G4 resolvase 1 binding**

Ramreddy Tippana^1^, Weikun Xiao^2^, Sua Myong^1,3,4,5^

1. Bioengineering Department, University of Illinois
2. University of California, Los Angeles
3. Biophysics and Computational Biology
4. Institute for Genomic Biology
5. Physics Frontier Center (Center of Physics for Living Cells), University of Illinois

Supplementary Figure 1

Supplementary Figure 2

Supplementary Figure 3

Supplementary Table 1

Supplementary Table 2


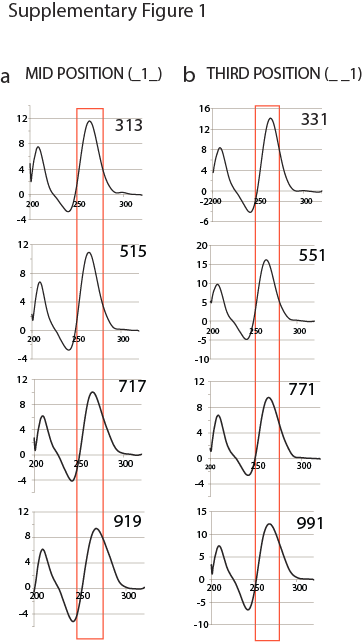


**Supplementary Figure 1** Position independent single nucleotide effect (a) CD spectrum of 313, 515, 717, 919 DNA constructs (single nucleotide in middle position) (b) CD spectrum of 331, 551, 771, 991 (single nucleotide in third position).


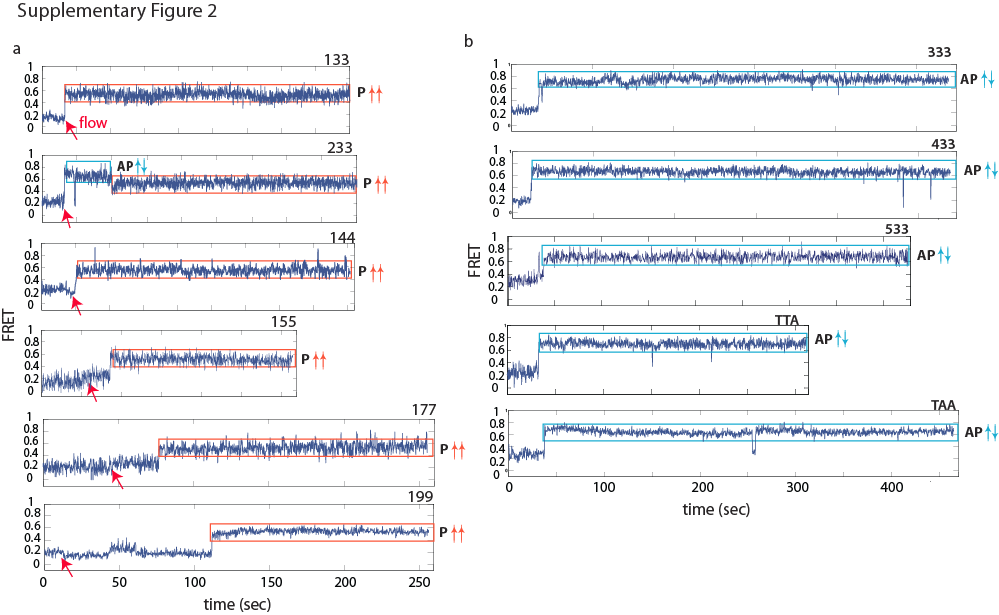


**Supplementary Figure 2** Long FRET traces (a) GQ constructs, 133, 233, 144, 155, 177, 199 remain folded in parallel conformation for extended period of time. (b) Small fraction of 333, 433, 533, TTA and TAA show stably folded state while majority of traces show dynamic inter-conversion between all three states.


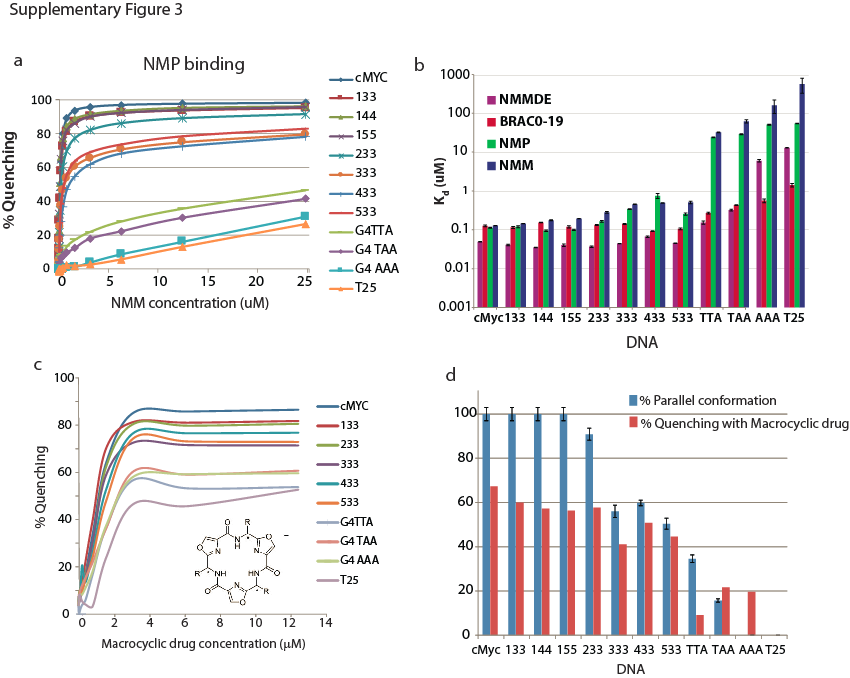


**Supplementary Figure 3** GQ ligand binding (a) NMP binding measured by Cy3 quenching in the same way as shown in Figure 6. (b) Binding dissociation constant, K_d_ measured for all DNAs. (c) Macrocyclic drug binding measured by Cy3 quenching. (d) % Parallel plotted with % quenching of macrocyclic drug shows poor correlation.

**Supplementary Table 1: Oligonucleotides used for G4 resolvase 1 purification and electrophoretic mobility shift assays**

| **Oligo name** | **Sequence 5’ to 3’** |
| --- | --- |
| **c-Myc T15** | TGG CGA CGG CAG CGA GGC GGG T GGG GA GGG T GGG TTT TTT TTT TTT TTT |
| **111 T15** | TGG CGA CGG CAG CGA GGC GGG T GGG T GGG T GGG TTT TTT TTT TTT TTT |
| **133 T15** | TGG CGA CGG CAG CGA GGC GGG T GGG TTT GGG TTT GGG TTT TTT TTT TTT TTT |
| **233 T15** | TGG CGA CGG CAG CGA GGC GGG TT GGG TTT GGG TTT GGG TTT TTT TTT TTT TTT |
| **333 T15** | TGG CGA CGG CAG CGA GGC GGG TTT GGG TTT GGG TTT GGG TTT TTT TTT TTT TTT |
| **433 T15** | TGG CGA CGG CAG CGA GGC GGG TTTT GGG TTT GGG TTT GGG TTT TTT TTT TTT TTT |
| **533 T15** | TGG CGA CGG CAG CGA GGC GGG TTTTT GGG TTT GGG TTT GGG TTT TTT TTT TTT TTT |
| **TTA T15** | TGG CGA CGG CAG CGA GGC GGG TTA GGG TTA GGG TTA GGG TTT TTT TTT TTT TTT |
| **TAA T15** | TGG CGA CGG CAG CGA GGC GGG TAA GGG TAA GGG TAA GGG TTT TTT TTT TTT TTT |
| **AAA T15** | TGG CGA CGG CAG CGA GGC GGG AAA GGG AAA GGG AAA GGG TTT TTT TTT TTT TTT |
| **5Cy518mer** | /5′Cy5/GCC TCG CTG CCG TCG CCA |
| **Biotin c-Myc 51 mer** | \|  \| /5BiodT/-GGC CGC TTA TGG GGA GGG TGG GGA GGG TGG GGA AGG TGG GGA GGA GAC TCA \| \| --- \| --- \| |

**Supplementary Table 2: Summary chart of GQ conformation and kinetic rates**


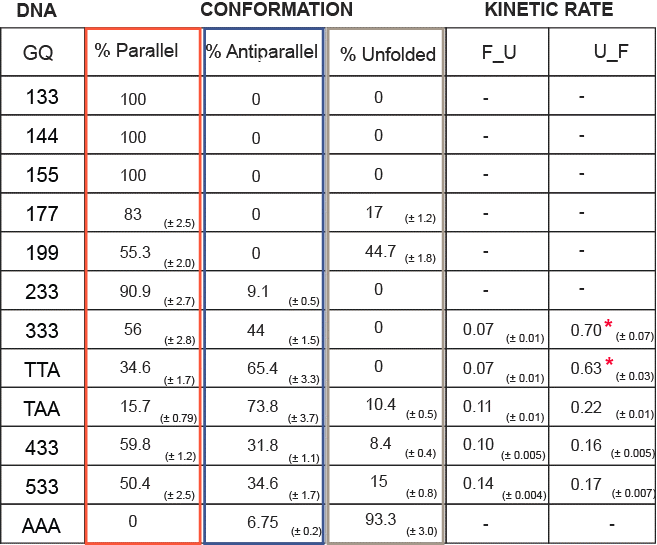


Kinetic rates of 333 (TTT), TTA, TAA, 433, 533 and AAA were calculated in the following way. The F_U (**un**folding rate) is an average between P_U and AP_U whereas the U_F (folding rate) is an average between U_P and U_AP.

The “*****” denotes the fast folding rate found in 333 (TTT) and TAA, as detailed in the manuscript.
